# The intermicrovillar adhesion complex in gut barrier function and inflammation

**DOI:** 10.37349/edd.2022.00006

**Published:** 2022-10-11

**Authors:** Bernadette Mödl, Katy Schmidt, Doris Moser, Robert Eferl

**Affiliations:** 1 Center for Cancer Research, Medical University of Vienna & Comprehensive Cancer Center, 1090 Vienna, Austria; 2 Division of Cell and Developmental Biology, Medical University of Vienna, 1090 Vienna, Austria; 3 Department of Cranio-Maxillofacial and Oral Surgery, Medical University of Vienna, 1090 Vienna, Austria

**Keywords:** Inflammatory bowel disease, colitis, microvilli, brush border, bacteria, microbiome, microbiota

## Abstract

The surface of intestinal epithelial cells is covered by the brush border, which consists of densely packed cellular extrusions called microvilli. Until recently, microvilli have not been known to be interconnected. In 2014, a protein complex, called the intermicrovillar adhesion complex (IMAC) which is located at the tips of the microvilli and responsible for the regular spatial organization of the brush border, was identified. Deletion of IMAC components such as cadherin-related family member-2 (CDHR2) in mice resulted in microvillus disorganization and fanning, a structural aberration that is also found in the brush border of patients with inflammatory bowel disease. The etiology of inflammatory bowel disease has been primarily associated with dysfunctional mucosal immunity, but the discovery of the IMAC may encourage theories of an epithelial origin. Here, possible effects of the brush border on the gut barrier function and intestinal inflammation are discussed proposing that the IMAC protects against inflammation through its microvillus cross-linking function.

## Introduction

Inflammatory bowel diseases (IBD) such as Crohn’s disease (CD) or ulcerative colitis (UC) are chronic inflammatory conditions of the gastrointestinal tract. Regions with a high prevalence of IBD (> 0.3% of inhabitants) are Europe and North America. However, the incidence is increasing in recently industrialized countries, making IBD an important contributor to total health expenditure [1]. Current knowledge suggests that IBD are driven by a complex interplay of genetic and environmental factors, dysregulated immunity, and a dysbiotic gut microbiome. This view is supported by genome-wide association studies that have identified a variety of IBD risk genes in microbe-sensing pathways, cytokine networks, inflammasome signaling, cell stress pathways, and mucosal barrier function [2]. However, it is currently unclear which factors are the cause or the consequence of the disease. In addition, identified risk genes are expressed in different types of intestinal epithelial cells as well as in innate and adaptive immune cells indicating complex epithelial-immune interactions [3]. It is likely that the significance of potential trigger factors is specific for each patient. This is underscored by the accumulation of evidence that IBD are more heterogeneous than the traditional UC-CD dichotomy. IBD can include many disease subtypes that can be distinguished based on the underlying trigger factors, treatment responses, and genetic associations [2]. Previous research has mainly focused on immune-mediated mechanisms in the development and maintenance of IBD [2]. However, more recent evidence suggests a significant contribution from the intestinal epithelium. The importance of the epithelial barrier is now undisputed and multiple pieces of evidence suggest that barrier defects alone are enough to trigger IBD [4].

## The intestinal brush border

**Figure 1:**
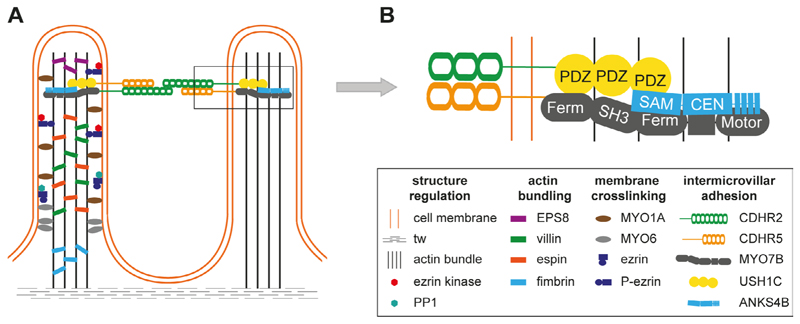
Structural and molecular organization of microvilli. A. Microvilli are stabilized by a core actin bundle, corresponding actin-bundling proteins (EPS8, villin, espin, fimbrin), and proteins connecting the actin bundle to the cell membrane [MYO1A, MYO6, ezrin]. The latter can be regulated by phosphorylation and dephosphorylation of ezrin by an ezrin kinase and protein phosphatase 1 (PP1); B. intermicrovillar adhesion is mediated by cadherin-related family member-2 (CDHR2) and CDHR5. Within the microvilli, MYO7B, Usher syndrome type I C (USH1C, harmonin), and ankyrin repeat and sterile alpha motif domain containing 4B (ANKS4B) interact with the intracellular portions of protocadherins and connect them to the actin bundle. The connections are mediated through multiple protein interaction motifs such as post-synaptic density protein 95, Drosophila disc large tumor suppressor, and zona occludens-1 protein (PDZ) domains in USH1C. CEN: central region; Ferm: 4.1 protein, ERM; SAM: sterile alpha motif; SH3: src homology 3

**Figure 2:**
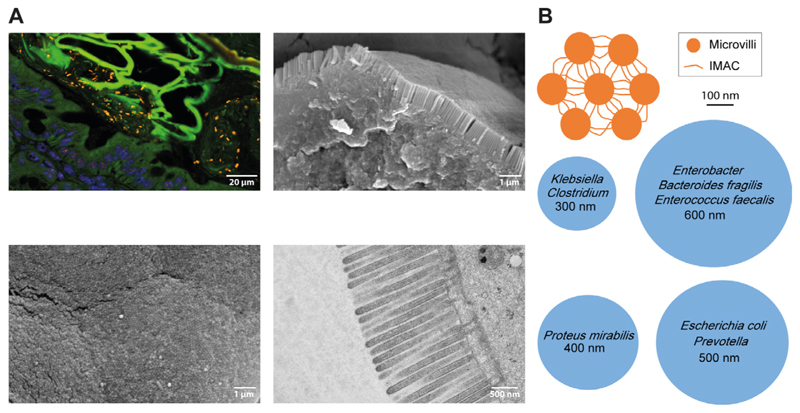
Tightly packed microvilli in the intestinal brush border may prevent access of bacteria to the apical cell membrane. A. Upper left image: spinning disc confocal image of a mouse colon section stained by fluorescence *in situ* hybridization (FISH) for the pan-bacterial probe EUB338 [27, 35] reveals many bacteria (orange rods and *cocci*) in the lumen of the large intestine that were unable to cross the epithelial barrier (nuclei in blue, green autofluorescence was used to demarcate cells). For FISH, intestinal tissues were fixed in Carnoy’s solution and 4 µm sections were stained with Cy3 end-labeled probes. Images were captured under a spinning disc fluorescence microscope (Olympus IXplore SpinSR). Lower left image: scanning electron microscopy (SEM) image showing microvillus tips en-face in the mouse small intestine. A regular hexagonal structure is evident. Upper right image: SEM image of a broken epithelial cell in the mouse small intestine showing the tightly packed brush border from aside. For SEM, tissue samples were washed with phosphate-buffered saline (PBS), fixed in 2.5% glutaraldehyde, and dehydrated in a graded ethanol series. Ethanol dehydration was followed by hexamethyldisilazane drying (HMDS, Sigma-Aldrich). Subsequently, samples were fixed to specimens mounts with double-faced adhesive carbon tape, gold-sputtered (Sputter Coater, ACE200, Leica Microsystems, Wetzlar, Germany), and examined in a scanning electron microscope (JSM 6310, JEOL Ltd^®^, Japan) at an acceleration voltage of 10–15 kV. Lower right image: transmission electron microscopy (TEM) image of microvilli on epithelial cells of the mouse small intestine. The tw below the cell membrane is also visible. For TEM, tissue samples were fixed in 2% paraformaldehyde and 2.5% glutaraldehyde. After washing in cacodylate, specimens were postfixed in 1% OsO_4_, dehydrated in a graded ethanol series, and infiltrated in Epon812. The 60 nm sections were imaged with an FEI Tecnai20 transmission electron microscope; B. top view schemes of microvilli connected by the intermicrovillar adhesion complex (IMAC, orange) and vertical cross-sections of common gut bacteria (blue). The bacterial diameter is indicated. The upper orange scheme and its dimensions were created based on the freeze-etch electron microscopy (EM) image of the mouse intestinal brush border in [15]

Microvilli of the intestinal brush border are regular cell extrusions stabilized by a core actin bundle and several other proteins (Figure 1). The first microvilli emerge early in enterocyte differentiation, and adjacent protrusions form clusters by adhesion between the distal tips. Additional microvilli are gradually incorporated into clusters and adjacent clusters fuse into larger structures. Eventually, a densely packed mature brush border without gaps has been formed, covering the entire surface of the differentiated enterocytes [5]. Microvilli are remarkably uniform in diameter (about 100 nm) and length (about 1–3 µm) and form highly ordered regular hexagonal structures (Figure 2A) [5]. The length depends on the region of the intestinal tract and can be modulated by nutrients such as glucose (high glucose concentration and glucose starvation increase and decrease microvillus length, respectively) [6]. The formation of microvilli is an energy-consuming process, as it requires a pushing force that deforms the cell membrane [7]. In the case of gut microvilli, polymerization of an actin bundle of about 30–40 actin filaments [8], which is stiff enough to push the cell membrane outward, could provide the force [5]. Indeed, microvillus length can be regulated by actin polymerization and depolymerization dynamics. The individual filaments of the actin bundle are held together by actin-bundling proteins villin, espin, and fimbrin (Figure 1A) [5]. The bundling proteins not only prevent disorganization of the microvillus actin core but may also be involved in early steps of membrane extrusion. It has been shown that exogenous villin can induce the formation of microvilli in cells that originally lack microvilli [9]. In addition, fimbrin anchors the microvilli roots to the terminal web (tw) [10], a contractile cell structure composed of cytokeratin that is located close to the inner lipid layer of the apical membrane. However, even mice with triple deletion of villin, espin, and fimbrin can form microvilli [11], suggesting that other actin-bundling proteins such as epidermal growth factor receptor kinase substrate 8 (EPS8) are involved in core actin stabilization [5]. Besides the actin-bundling proteins, several proteins of the ezrin, radixin, moesin (ERM), and myosin (MYO) families are required for microvillus stabilization as they connect the actin core to the lipid bilayers of the microvilli [5]. Otherwise, the membrane extrusions would go to their lowest energy level and melt together. The proteins that prevent membrane coalescence of the densely packed microvilli include MYO1A, MYO6, and ezrin [5]. Individual deletion of all these proteins in mice resulted in the fusion of microvilli [12–14].

## The IMAC

The IMAC is a protein complex that cross-links microvilli of the intestinal brush border (Figure 1). The cross-links long overlooked in electron micrographs of intestinal epithelial cells were eventually discovered in CACO-2_BBE_ cells (CVCL_1096) [15]. These human colon adenocarcinoma cells form a brush border when grown to confluency *in vitro* [15]. Using this cell model, adhesions between the apical microvillus tips consisting of a transmembrane protein complex were recognized. The extracellular part of the IMAC consists of the extracellular domains of protocadherins CDHR2 and CDHR5. Their intracellular domains interact with cytoplasmic proteins, including USH1C, the actin-based motor MYO7B, and the ankyrin repeat protein ANKS4B [5, 15, 16], that anchor the IMAC to the core actin bundle of microvilli [5, 15]. Deletion of protocadherins in CACO-2_BBE_ cells revealed that without a functional IMAC, the brush border looks like a coniferous forest with windbreaks. In addition, microvillus shortening has been observed, but it is not known how the IMAC regulates microvillus length [15]. This indicates that the main function of the IMAC is microvillus bridging to provide regular spatial organization and uniform microvillus length in the brush border [5, 15]. Structural consequences of IMAC ablation were demonstrated *in vivo* in mice lacking CDHR2 in the gut epithelium. Microvilli in the brush border were shortened, disorganized, and lost their circularity. The other IMAC components CDHR5, USH1C, and MYO7B were not properly localized at the microvillus tip and the expression of several apical markers such as intestinal alkaline phosphatase (IAP) was reduced [17].

## The brush border and the IMAC in intestinal barrier function and IBD

Various functions have been ascribed to the brush border. The microvilli increase the surface area of small intestinal epithelial cells by 9-fold to 16-fold [18], providing more space for membrane-associated molecules involved in the uptake of nutrients [19] or defense against pathogens [20]. According to the diffusion barrier model, nutrient uptake can be controlled by modulating microvillus length. When nutrients are taken up by transporters in the microvillus tips, they must diffuse throughout the microvillus to reach the cytoplasm. The bundle of actin in the microvillus can provide a diffusion barrier that depends on the length of the microvillus [6]. Whether the brush border plays a role in the barrier function of the intestine is currently unknown. The maintenance of the mucosal barrier relies on the production of mucus and several antimicrobial peptides such as defensins, cathelicidins, or regenerating gene III/α/β/γ peptides by goblet cells and Paneth cells as well as on tight junctions that seal the epithelial cell layers [21]. Low molecular weight compounds can cross the tight junctions via the paracellular pathway between two cells, but they are impermeable for high molecular weight compounds. Under physiological and non-disease conditions, only few (if any) bacteria may cross the intestinal epithelium (Figure 2A). When bacterial transmission occurs, it is mainly via the transcellular route through endocytic mechanisms [21, 22]. However, there have also been rare reports of paracellular bacterial invasion [22, 23]. There is no direct experimental evidence that the IMAC prevents bacterial transmission and is involved in inflammatory bowel disease. Spontaneous colitis has not been reported in mice with deletion of CDHR2 in intestinal epithelial cells. This indicates that loss of IMAC is not a primary trigger of intestinal inflammation. However, it is conceivable that the protective effect of the mucus layer is sufficient to prevent bacterial transmission independent of brush border disorganization. In this case, experimental disruption of the mucus layer would reveal the importance of the IMAC in colitis protection. However, IMAC-ablated mice have not yet been bred to mice lacking a proper mucus layer, such as mucin 2 (MUC2)-deficient mice. Enhanced adhesion and internalization of bacteria by intestinal epithelial cells have been observed in patients with IBD [24, 25]. The inflamed epithelia showed fanning of microvilli [26], allowing bacteria to gain access to cholesterol-rich lipid rafts and caveolae at the base of the intermicrovillous cleft. Under this condition, even non-invasive mucosal bacteria (which do not have a specialized molecular machinery for cell invasion) could potentially hijack lipid raft/caveolae-mediated endocytosis for cell penetration [27]. In a recent study, altered gene clusters were analyzed in tissue samples of CD patients [26]. Interestingly, a gene cluster that is associated with the brush border of enterocytes and contains CDHR2 and CDHR5, was identified in almost all patients. Electron micrographs showed microvillus aberrations in patients, the severity of which correlated with the expression of the brush border cluster genes. The expression also correlated with the endoscopy score of CD patients in the UNITI-2 trial, which examined the effects of ustekinumab on CD patients. The authors suggested that microvillus aberrations contribute to the course or even trigger the disease in CD patients [26].

## Possible effects of IMAC disorganization on bacteria

**Figure 3:**
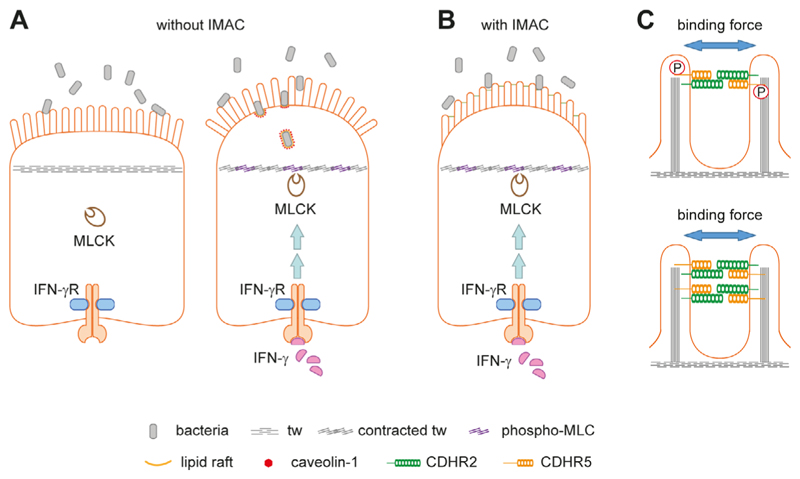
The arc model and how the IMAC could prevent fanning of microvilli. A. Schemes of intestinal epithelial cells without (left) and with inflammation (right). The brush border serves as a physical barrier for luminal microbes (left). During inflammation, IFN-γ can activate MLCK leading to aberrant MLC phosphorylation and contraction of the tw (right). The latter results in tight junction disruption (not indicated), arc formation of the apical cell membrane, and brush border fanning. The enlarged intermicrovillus clefts allow bacterial penetration and internalization through cholesterol-rich lipid raft/caveolin-1-dependent endocytosis. This panel was created based on [21]; B. the IMAC was not included in the original arc model shown in Figure 2A. Microvillus cross-linking might prevent IFN-γ-induced brush border fanning; C. two possibilities how the IMAC binding force for microvilli could be modulated. The intracellular parts of protocadherins contain several domains for protein-protein interaction and putative phosphorylation sites. Phosphorylation by corresponding kinases could result in a conformational change of protocadherins (not indicated) that increases or reduces the binding force (upper scheme). Alternatively, higher expression of IMAC components could increase the number of IMAC complexes between microvilli thereby increasing the binding force (lower scheme). P: phosphate

If bacteria gain access to the brush border, densely packed microvilli of uniform length would be required to prevent the formation of gaps suitable for bacterial entry and lipid raft/caveolae-mediated endocytosis [21]. The hexagonal arrangement of the microvilli provokes the formation of radial structures of multiple IMAC complexes, which could provide a physical barrier against bacterial invasion. Neighboring microvilli are connected by multiple IMAC complexes, making this physical barrier even more rigid. However, loss of connections or loss of single microvilli would create gaps wide enough for bacteria to enter, at least for such bacterial species with a small diameter (Figure 2B). It is also conceivable that a disorganized brush border creates a separate niche for bacteria, which can alter the growth environment of the gut microbiota and lead to dysbiosis. The brush border has been shown to affect bacterial growth in the intestinal lumen through the production of lumenal vesicles containing IAP, which can detoxify lipopolysaccharide. The vesicles bud from the microvillus tips and can prevent bacteria from adhering to the intestinal epithelium. Their production is induced by bacterial pathogens, indicating an active defense mechanism [28]. IMAC ablation and microvillus disorganization could affect lumenal vesicle budding from the microvillus tips, thereby reducing bacterial defenses. Interestingly, paracellular and transcellular leakage are frequently co-occurring conditions in IBD. Inflammatory cytokines such as interferon-gamma (IFN-γ) and tumor necrosis factor-alpha (TNF-α) can affect the paracellular pathway via modulating tight junction protein distribution [4] but their role in transcellular leakage remains unclear. It has been shown that IFN-γ can induce aberrant phosphorylation of MYO light chain (MLC) by MYO light chain kinase (MLCK) in the tw of murine intestinal epithelial cells, resulting in tw contraction, disruption of tight junctions, and bacterial transmission. The latter was observed even before tight junction disruption. Correspondingly, an arc model for transcellular leakage has been proposed (Figure 3A) [21, 27]. The model asserts that upon contraction of the tw, the apical membrane forms an arc that exerts a fanning force on the microvilli. Without the IMAC, fanning would occur, creating gaps for bacteria (Figure 3A). Microvillus cross-linking by the IMAC could prevent arc-induced brush border fanning and gap formation (Figure 3B). Increased phosphorylation of MLC has also been observed in IBD, suggesting that arcing occurs in the inflamed mucosa of patients. It is currently believed that the IMAC is a rigid and unchanging structure for microvillus cross-linking, but it is possible that cross-linking can be modulated by phosphorylation of protocadherins or by increased expression of IMAC components (Figure 3C).

## Conclusion and outlook

Invasion of the epithelium by non-invasive commensal bacteria could make them pathobionts and increase susceptibility to colitis [21]. Adherence and penetration of non-invasive bacteria have been documented frequently in patients with IBD [25, 29] and celiac disease [30]. Penetration might occur due to microvillar fanning, which might be exacerbated by the action of IFN-γ [27, 31]. Although a protective role of the brush border and the IMAC in intestinal inflammation have yet to be demonstrated experimentally, it is highly likely that the regular organization of the densely packed microvilli is needed to prevent bacterial invasion. Drugs that induce the expression of IMAC components in intestinal epithelial cells could strengthen mucosal barrier function and provide a therapeutic regiment for IBD. The anti-inflammatory drug mesalazine is often used to treat IBD patients [32]. The exact mechanism of action of mesalazine is unknown, but it is a potent inducer of CDHR5 expression in intestinal cell lines [33, 34]. Although its ability to induce CDHR5 *in vivo* has yet to be established, mesalazine may hold promise as a treatment for patients with a compromised gut barrier. Furthermore, the identification of drugs that enhance the microvillus binding power of the IMAC would provide a new opportunity to improve barrier function.

## Data Availability

Not applicable.

## Abbreviations

ANKS4B: ankyrin repeat and sterile alpha motif domain containing 4B
CD: Crohn’s disease
CDHR2: cadherin-related family member-2
EPS8: epidermal growth factor receptor kinase substrate 8
IBD: inflammatory bowel diseases
IFN-γ: interferon-gamma
IMAC: intermicrovillar adhesion complex
MLC: myosin light chain
MLCK: myosin light chain kinase
MYO: myosin
SEM: scanning electron microscopy
tw: terminal web
USH1C: Usher syndrome type I C
